# Stochastic Expansions Maintain the Clonal Stability of CD8^+^ T Cell Populations Undergoing Memory Inflation Driven by Murine Cytomegalovirus

**DOI:** 10.4049/jimmunol.1900455

**Published:** 2019-12-17

**Authors:** Corinne J. Smith, Vanessa Venturi, Maire F. Quigley, Holly Turula, Emma Gostick, Kristin Ladell, Brenna J. Hill, Danielle Himelfarb, Kylie M. Quinn, Hui Yee Greenaway, Thurston H. Y. Dang, Robert A. Seder, Daniel C. Douek, Ann B. Hill, Miles P. Davenport, David A. Price, Christopher M. Snyder

**Affiliations:** * Department of Microbiology and Immunology, Sidney Kimmel Cancer Center, Thomas Jefferson University, Philadelphia, PA 19107;; † Infection Analytics Program, Kirby Institute for Infection and Immunity, UNSW Sydney, Sydney, New South Wales 2052, Australia;; ‡ Human Immunology Section, Vaccine Research Center, National Institute of Allergy and Infectious Diseases, National Institutes of Health, Bethesda, MD 20892;; § Division of Infection and Immunity, Cardiff University School of Medicine, University Hospital of Wales, Cardiff CF14 4XN, United Kingdom;; ¶ Cellular Immunology Laboratory, Vaccine Research Center, National Institute of Allergy and Infectious Diseases, National Institutes of Health, Bethesda, MD 20892; and; ‖ Department of Molecular Microbiology and Immunology, Oregon Health & Science University, Portland, OR 97239

## Abstract

CMV is an obligate and persistent intracellular pathogen that continually drives the production of highly differentiated virus-specific CD8^+^ T cells in an Ag-dependent manner, a phenomenon known as memory inflation. Extensive proliferation is required to generate and maintain inflationary CD8^+^ T cell populations, which are counterintuitively short-lived and typically exposed to limited amounts of Ag during the chronic phase of infection. An apparent discrepancy therefore exists between the magnitude of expansion and the requirement for ongoing immunogenic stimulation. To address this issue, we explored the clonal dynamics of memory inflation. First, we tracked congenically marked OT-I cell populations in recipient mice infected with murine CMV (MCMV) expressing the cognate Ag OVA. Irrespective of numerical dominance, stochastic expansions were observed in each population, such that dominant and subdominant OT-I cells were maintained at stable frequencies over time. Second, we characterized endogenous CD8^+^ T cell populations specific for two classic inflationary epitopes, M38 and IE3. Multiple clonotypes simultaneously underwent Ag-driven proliferation during latent infection with MCMV. In addition, the corresponding CD8^+^ T cell repertoires were stable over time and dominated by persistent clonotypes, many of which also occurred in more than one mouse. Collectively, these data suggest that stochastic encounters with Ag occur frequently enough to maintain oligoclonal populations of inflationary CD8^+^ T cells, despite intrinsic constraints on epitope display at individual sites of infection with MCMV.

## Key Points

- Clonal stability is a feature of memory inflation.
- Stochastic expansions maintain clonal stability during memory inflation.
- Persistent clonotypes are often public in the context of memory inflation.

## Introduction

Cytomegalovirus is a ubiquitous betaherpesvirus that persists in a state of latency. Acute infection is usually asymptomatic in immunocompetent hosts, but lifelong immune surveillance is required to suppress viral reactivation and prevent disease (1). In the latent phase of infection, viral reactivation occurs in an infrequent and sporadic manner, such that a vast majority of infected cells harbor transcriptionally silent virus at any one time (2–4). However, the process of viral reactivation in individual cells is highly coordinated and follows a predictable course, with stepwise expression of immediate-early, early, and late genes (3). Epitopes derived from immediate-early proteins in particular form key targets for CD8^+^ T cells, enabling immune-mediated termination of the viral lifecycle before the release of mature virions, at least in mice infected with murine CMV (MCMV) (5). As a consequence of viral reactivation events, the immune system is periodically exposed to a largely constant set of Ags. In turn, recurrent stimulation drives the amplification and maintenance of various CMV-specific CD8^+^ T cell populations, a phenomenon known as memory inflation (6–10).

Memory inflation in mice depends on Ag presentation by infected nonhematopoietic cells (11, 12). However, viral transcripts are rare during the latent phase of infection (4, 13), leading to competition among virus-specific CD8^+^ T cells for the same (14) or different epitopes (15). Similar phenomena occur in humans. Accordingly, human CMV (HCMV)–specific CD8^+^ T cell populations are generally oligoclonal (16–22) and express TCRs that display conserved patterns of amino acid use across the CDR3 loop (20, 23, 24), many of which are shared among individuals and therefore classified as “public” (16–20, 23–25). These features are so pervasive in fact that infection status (26) and even the specificity of certain public sequences (27) can be determined from bulk peripheral repertoire datasets, reflecting consistent patterns of clonal selection governed at the level of Ag engagement by cognate TCRs (16, 17, 21–23).

CD8^+^ T cells that undergo memory inflation typically display a highly differentiated phenotype (CD127^−^KLRG1^+^) (8, 28, 29). In humans and mice, these cells are limited in terms of proliferative capacity and survival, with a *t*_1/2_ of 45–60 d (29, 30). Earlier work suggested that such effector and/or effector-memory cells are continually replenished from a much smaller, less differentiated pool of memory CD8^+^ T cells (CD127^+^KLRG1^−^) (11, 31), driven through multiple rounds of division by recurrent Ag exposure (13, 29). Accordingly, memory inflation seems to be founded on a process of competition for access to limited amounts of cognate Ag, in which success leads to preferential expansion of the “fittest” clones.

In this study, we addressed two unresolved issues in the field of memory inflation, namely whether numerical dominance per se provides a selection advantage over time and to what extent clonal dynamics impact the overall constitution of inflationary CD8^+^ T cell populations. Our findings indicate that stochastic encounters with Ag drive the occasional emergence and expansion of rare clonotypes within otherwise highly stable CD8^+^ T cell populations specific for inflationary epitopes derived from MCMV.

## Materials and Methods

### Mice

Parental mouse strains were purchased from the Jackson Laboratory. C57BL/6 mice were used for direct infections. CD45.1 congenic mice (B6.SJL-*Ptprc^a^ Pepc^b^*/BoyJ) and OT-I mice [C57BL/6-Tg(TcraTcrb)1100Mjb/J] were used for adoptive transfers. CD45.1^+^ OT-I mice and CD45.1^+^/CD45.2^+^ OT-I mice were bred in house. All experimental procedures were approved by the Institutional Biosafety Committee and the Institutional Animal Care and Use Committee at Oregon Health & Science University.

### Viruses

Mice were infected i.p. with 2 × 10^5^ PFU of MCMV. Endogenous CD8^+^ T cell responses were analyzed postinfection with strain K181. Adoptive transfer experiments with OT-I cells were performed postinfection with strain SL8-015 (MCMV-OVA). Viruses were grown and titrated on M2-10B4 cells (32).

### Flow cytometry

Lymphocytes were isolated from organs as described previously (13). Peripheral blood samples and splenocytes were analyzed immediately or mixed with 10% DMSO and cryopreserved at −80°C. Fluorochrome-labeled tetrameric complexes of SSPPMFRV/H-2K^b^ (M38, residues 316–323) and RALEYKNL/H-2K^b^ (IE3, residues 416–423) were generated in house (17). Directly conjugated mAbs were purchased from commercial vendors (BD Biosciences or BioLegend). Staining procedures were described previously (29). OT-I cells were identified using anti-CD45.1 (clone A20), anti-CD45.2 (clone 104), and anti-TCR Vα2 (clone B20.1). Differentiation and proliferation were assessed using anti-CD8a (clone 53-6.7), anti-CD127 (clone A7R34), anti-KLRG1 (clone 2F1), and anti-Ki67 (clone B56). Repertoires were characterized at the protein level using an Anti-Mouse TCR Vβ Screening Panel (BD Biosciences). Data were acquired using an LSR II flow cytometer (BD Biosciences) and analyzed with FlowJo software versions 9.3 and 10.2 (Tree Star).

### Clonotype identification

Frozen vials of peripheral blood or splenocytes were thawed at 37°C. RBCs were lyzed using ACK Lysing Buffer (Thermo Fisher Scientific). Lymphocytes were then washed twice in RPMI 1640 medium supplemented with 10% FCS (R10), stained with fluorochrome-labeled tetrameric complexes of SSPPMFRV/H-2K^b^ and RALEYKNL/H-2K^b^ for 10 min at 37°C, washed again twice in R10, and stained with anti-CD3 (clone 17-A2), anti-CD4 (clone RM4-5), and anti-CD8a (clone 53-6.7) for 20 min at 4°C. Dead cells were excluded using a LIVE/DEAD Fixable Violet Dead Cell Stain Kit (Thermo Fisher Scientific). Viable Ag-specific CD8^+^ T cells (amine reactive dye^−^, CD3^+^, CD8^+^, and either M38 or IE3 tetramer^+^) were sorted at >98% purity using a modified FACSAria flow cytometer (BD Biosciences). Aliquots of 1000 sorted cells were collected directly into Screw Cap Micro Tubes (Sarstedt) containing 150 μl of RNAlater (Thermo Fisher Scientific). Unbiased molecular determination of all expressed *TRB* gene products was conducted using a template-switch anchored RT-PCR as described previously (17, 33, 34).

### Repertoire analysis

Each TCRβ sequence was aligned sequentially with the best-matched *TRBV* gene, followed by the best-matched *TRBJ* gene and the best-matched *TRBD* gene, using the IMGT reference alleles for *Mus musculus* (35). The CDR3β sequence was then identified inclusively between the conserved cysteine in the Vβ region and the conserved phenylalanine in the Jβ region. The minimum number of nucleotide additions required to produce a CDR3β sequence was determined by germline matching to the *TRBV* and *TRBJ* genes, followed by the *TRBD* gene (minimum *n* = 2 nt). Palindromic nucleotides were allowed at the 3′ end of the *TRBV* gene, the 5′ and 3′ ends of the *TRBD* gene, and the 5′ end of the *TRBJ* gene (maximum *n* = 6 nt). Junctional nucleotides that could not be assigned to germline genes were considered to be nucleotide additions.

Clonotype identity was defined at the nucleotide level (*TRBV* and *TRBJ* gene use and CDR3β nucleotide sequence). Persistent clonotypes were defined as those observed at more than one timepoint in peripheral blood samples obtained from a given mouse (days 195, 230, 265, and 302 postinfection with MCMV). Public clonotypes were defined at the amino acid level on the basis of exact sequence matches in more than one mouse, irrespective of prevalence and recurrence across all samples obtained from any one mouse (*n* = 6). Repertoire diversity was evaluated using the Simpson diversity index (36), and repertoire similarity was evaluated using the Morisita–Horn similarity index (37). These relative measures of diversity and similarity range in value from 0 (minimum diversity/similarity) to 1 (maximum diversity/similarity). The corresponding diversity and similarity indices were estimated as median values from 10,000 random draws of 32 sequences per repertoire to account for differences in sampling at the molecular level (36, 37). All diversity and similarity analyses were performed using MATLAB (MathWorks).

### Statistics

Statistical tests were performed using Prism version 8.1.0 (GraphPad Software).

## Results

### Cell division is a feature of memory inflation

**FIGURE 1.**
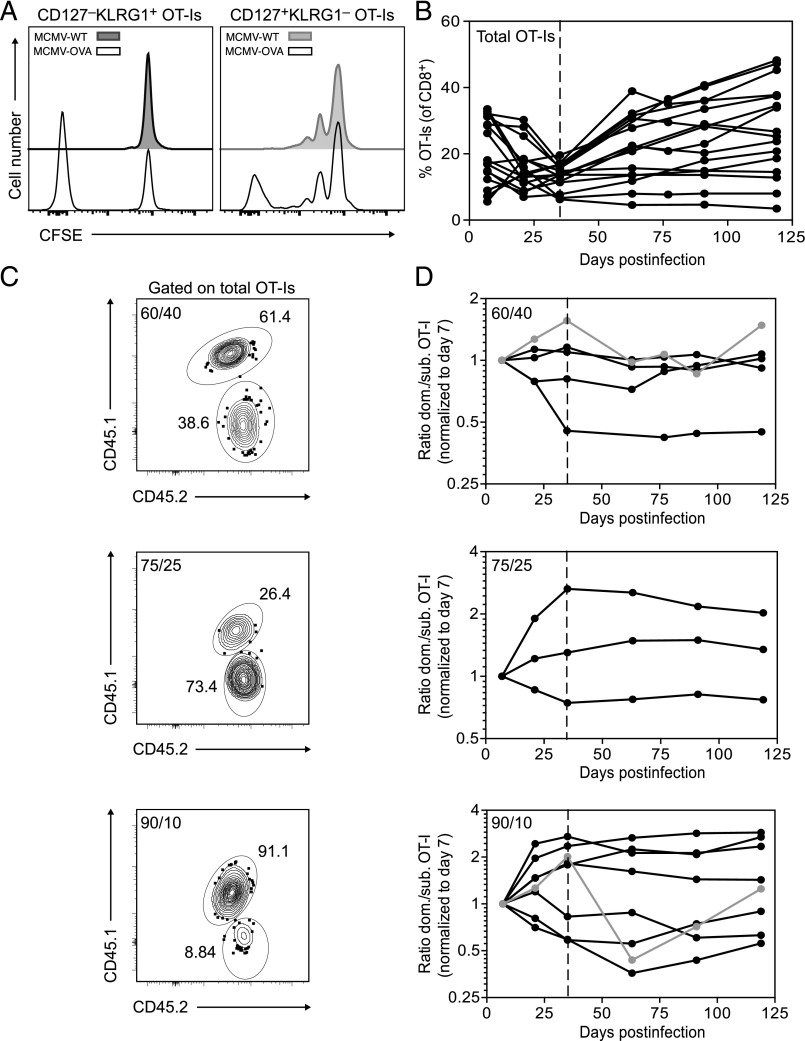
Clonal dominance does not impart a selection advantage during infection with MCMV. (**A**) Naive mice were seeded with congenically marked OT-I cells and infected with MCMV-OVA. After 120 d, CD8^+^ T cells were harvested from the spleens of primary recipient mice, labeled with CFSE, and transferred into secondary recipient mice latently infected with either wild-type MCMV (MCMV-WT) or MCMV-OVA. Flow cytometry histograms show CD127^−^KLRG1^+^ OT-I cells (left) and CD127^+^KLRG1^−^ OT-I cells (right) in the spleen 2 wk after transfer. Data were concatenated from three independent experiments. (**B**–**D**) Naive CD45.1 mice were seeded with mixtures of CD45.1^+^/CD45.2^+^ and CD45.2^+^ OT-I cells (total *n* = 1000) at different ratios (60:40, 75:25, and 90:10) and infected with MCMV-OVA. (B) Frequency of OT-I cells among CD8^+^ T cells in the peripheral blood over time. (C) Representative flow cytometry plots showing congenically marked OT-I cells in the peripheral blood on day 7 postinfection. Inset numbers indicate percentages. Top, Initial ratio 60:40. Middle, Initial ratio 75:25. Bottom, Initial ratio 90:10. (D) Ratio of congenically marked OT-I cells in the peripheral blood over time normalized to the dominant/subdominant ratio on day 7 postinfection. Top, Initial ratio 60:40. Middle, Initial ratio 75:25. Bottom, Initial ratio 90:10. Gray lines and symbols indicate mice with substantial fluctuations in the OT-I cell ratio during chronic infection (after day 35).

The production of highly differentiated effector and/or effector-memory CD8^+^ T cells is thought to require extensive proliferation (29). To confirm this earlier finding, we seeded naive mice with OT-I cells, which recognize the H-2K^b^–restricted OVA-derived epitope SIINFEKL (residues 257–264) via a transgenic TCR. Primary recipient mice were then infected with a recombinant MCMV expressing OVA (MCMV-OVA). Splenocytes were harvested 4 mo postinfection, labeled with CFSE, and transferred into secondary recipient mice latently infected with either wild-type MCMV or MCMV-OVA. After 2 wk, CD127^−^KLRG1^+^ OT-I cells in the spleens of secondary recipient mice infected with wild-type MCMV remained fully loaded with CFSE, indicating a lack of division (Fig. 1A, left panel), whereas the corresponding CD127^+^KLRG1^−^ OT-I cells displayed intermediate levels of CFSE, consistent with one or two rounds of homeostatic division (Fig. 1A, right panel). In contrast, CD127^−^KLRG1^+^ OT-1 cells recovered at the same timepoint from the spleens of secondary recipient mice infected with MCMV-OVA either remained fully loaded with CFSE or contained no trace of CFSE, indicating more than six rounds of division (Fig. 1A, left panel). Ongoing exposure to Ag was therefore required for the emergence of CFSE^low^CD127^−^KLRG1^+^ OT-I cells, which likely originated from a less differentiated memory compartment, given the limited proliferative capacity of CD127^−^KLRG1^+^ MCMV-specific CD8^+^ T cells (31).

### Stochastic expansions maintain clonal stability during memory inflation

**FIGURE 2.**
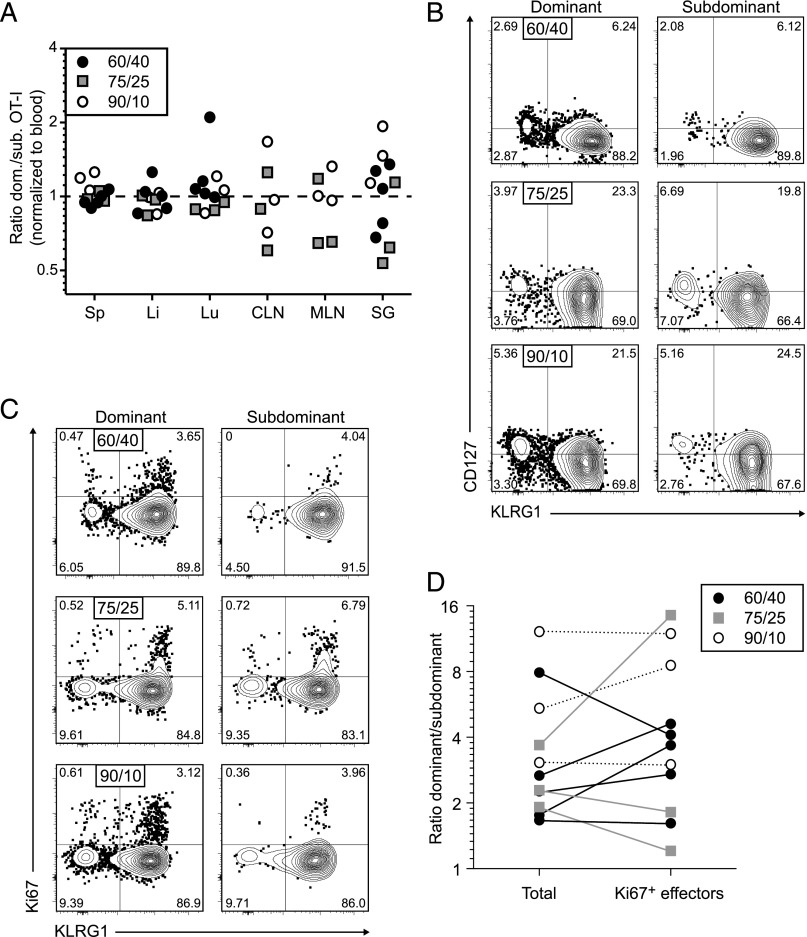
Stochastic expansions occur irrespective of clonal dominance hierarchies during infection with MCMV. Naive CD45.1 mice were seeded with mixtures of CD45.1^+^/CD45.2^+^ and CD45.2^+^ OT-I cells (total *n* = 1000) and sacrificed on day 120 after subsequent infection with MCMV-OVA. (**A**) Ratio of congenically marked OT-I cells in the spleen (Sp), liver (Li), lung (Lu), cervical lymph nodes (CLN), mediastinal lymph nodes (MLN), and salivary glands (SG) normalized to the dominant/subdominant ratio in the peripheral blood. Symbols denote initial ratios as indicated in the key. (**B**) Representative flow cytometry plots showing expression of CD127 and KLRG1 among dominant and subdominant OT-I cell populations in the spleen. Quadrant numbers indicate percentages. Top, Initial ratio 60:40. Middle, Initial ratio 75:25. Bottom, Initial ratio 90:10. (**C**) Representative flow cytometry plots showing expression of Ki67 and KLRG1 among dominant and subdominant OT-I cell populations in the spleen. Quadrant numbers indicate percentages. Top, Initial ratio 60:40. Middle, Initial ratio 75:25. Bottom, Initial ratio 90:10. (**D**) Ratio of congenically marked OT-I cells in the spleen comparing all events and Ki67^+^ events. Lines connect data points from the same mouse. Symbols denote initial ratios as indicated in the key.

To assess how these proliferation events might shape the process of memory inflation, we seeded naive mice with mixtures of two congenically marked OT-I cell populations at different ratios (60:40, 75:25, and 90:10) and infected the recipients with MCMV-OVA (Fig. 1B–D). In a vast majority of mice, the circulating OT-I cell population as a whole expanded progressively after transfer, emulating a classic inflationary profile (Fig. 1B). The relative frequencies of each congenically marked population fluctuated during early infection, a period of transient decline for the initial OT-I cell expansions, and then stabilized at various levels during chronic infection, although further oscillations were apparent in two mice (Fig. 1B, 1D). These equilibrium frequencies were largely recapitulated in the spleen and other heavily vascularized organs (Fig. 2A). However, the ratios of congenically marked OT-I cells varied to a greater extent around similar mean values in the lymph nodes and salivary glands compared with the spleen, liver, and lungs during chronic infection, potentially reflecting anatomical compartmentalization and the hematogenous nature of memory inflation (13).

At each timepoint during chronic infection, the dominant and subdominant populations of circulating OT-I cells were highly differentiated (CD127^−^KLRG1^+^), consistent with repeated exposure to cognate Ag (Fig. 2B). A fraction of cells in each population also expressed Ki67, a marker of cell proliferation (Fig. 2C). Similar results were obtained using OT-I cells isolated from various organs (data not shown). However, Ki67^+^ events were not always uniformly distributed among the dominant and subdominant populations, such that the “dividing” ratios were often skewed relative to the “total” ratios of congenically marked OT-I cells (Fig. 2D). In other words, the relative frequencies of OT-I cells undergoing active proliferation in the dominant and subdominant populations at a particular moment in time were not consistently or simply associated with the overall frequencies of OT-I cells in the dominant and subdominant populations, indicating that numerical dominance per se was not sufficient to gain a division advantage in vivo. Clonal expansions therefore occurred in a stochastic manner, likely reflecting chance encounters with cognate Ag, which by extension occurred frequently enough to maintain the dominant and subdominant populations of OT-I cells (Fig. 1D).

### Repertoire bias is common during memory inflation

**FIGURE 3.**
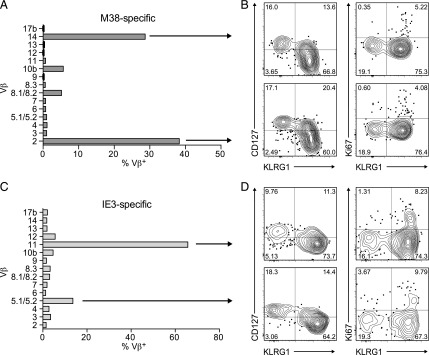
Distinct clonotypes undergo stochastic expansions contemporaneously during infection with MCMV. Splenocytes were stained with fluorochrome-labeled tetrameric complexes of SSPPMFRV/H-2K^b^ (M38) and RALEYKNL/H-2K^b^ (IE3) and a panel of mAbs specific for various Vβ segments on day 120 postinfection with MCMV. (**A**) Representative Vβ expression profile among M38-specific CD8^+^ T cells from one mouse. (**B**) Representative flow cytometry plots showing expression of CD127 and KLRG1 (left) or Ki67 and KLRG1 (right) among the Vβ-defined M38-specific CD8^+^ T cells depicted in (A). Quadrant numbers indicate percentages. (**C**) Representative Vβ expression profile among IE3-specific CD8^+^ T cells from one mouse. (**D**) Representative flow cytometry plots showing expression of CD127 and KLRG1 (left) or Ki67 and KLRG1 (right) among the Vβ-defined IE3-specific CD8^+^ T cells depicted in (C). Quadrant numbers indicate percentages. Total *n* = 5 mice.

To assess the impact of these processes on endogenous MCMV-specific CD8^+^ T cell populations, which incorporate naturally selected clonotypes expressing distinct TCRs, we used a panel of Vβ-specific mAbs in conjunction with fluorochrome-labeled tetrameric complexes representing the inflationary epitopes SSPPMFRV/H-2K^b^ (M38, residues 316–323) and RALEYKNL/H-2K^b^ (IE3, residues 416–423). Each of the corresponding inflationary CD8^+^ T cell populations displayed markedly restricted Vβ expression among splenocytes isolated on day 120 postinfection with MCMV (Fig. 3A, 3C). In particular, M38-specific CD8^+^ T cells typically expressed Vβ2 (TRBV1), whereas IE3-specific CD8^+^ T cells typically expressed Vβ11 (TRBV16). Akin to the OT-I data (Fig. 2B–D), Ki67^+^ events were present at comparable frequencies among the dominant and subdominant Vβ-defined CD8^+^ T cell populations specific for M38 or IE3, which also displayed largely similar phenotypes characterized by a general lack of CD127 and widespread expression of KLRG1 (Fig. 3B, 3D).

**FIGURE 4.**
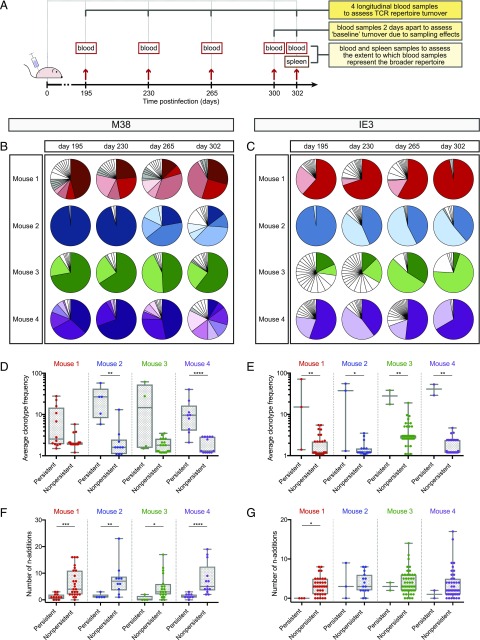
Inflationary CD8^+^ T cell repertoires are clonotypically stable during infection with MCMV. (**A**) Schematic representation of the sampling strategy. (**B** and **C**) Molecular analysis of M38-specific (B) or IE3-specific CD8^+^ T cell clonotypes (C) in the peripheral blood over time postinfection with MCMV. Each pie segment corresponds to a unique sequence. Colored segments indicate persistent clonotypes that occupied ≥5% of the entire repertoire at any one timepoint. Gray segments indicate persistent clonotypes that occupied <5% of the entire repertoire at all timepoints. White segments indicate nonpersistent clonotypes. (**D** and **E**) Average frequency of persistent and nonpersistent M38-specific (D) or IE3-specific CD8^+^ T cell clonotypes (E) across all timepoints. (**F** and **G**) Minimum number of nucleotide additions required to generate persistent and nonpersistent M38-specific (F) or IE3-specific CD8^+^ T cell clonotypes (G) across all timepoints. *p < 0.05, **p < 0.01, ***p < 0.001, ****p < 0.0001 (Mann–Whitney *U* test).

In further experiments, we used an unbiased molecular approach to track individual clonotypes in flow-sorted populations of inflationary CD8^+^ T cells isolated directly ex vivo from peripheral blood samples obtained on days 195, 230, 265, 300, and 302 postinfection with MCMV (17, 33, 34). Splenocytes were analyzed in parallel at the final timepoint (Fig. 4A). Data metrics are summarized in Supplemental Table IA. In line with our findings at the protein level (Fig. 3A, 3C), M38-specific CD8^+^ T cells typically expressed TRBV1 (Supplemental Table IB), whereas IE3-specific CD8^+^ T cells typically expressed TRBV16 (Supplemental Table IC).

### Persistent clonotypes are ubiquitous during memory inflation

**FIGURE 5.**
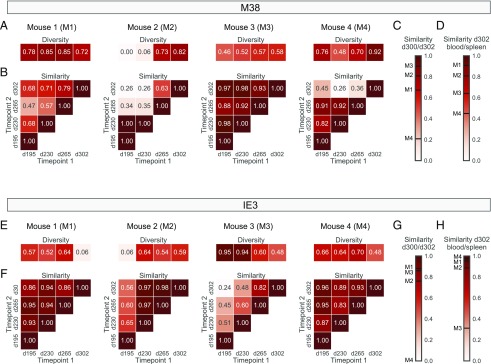
Inflationary CD8^+^ T cell repertoires generally retain population-level characteristics during infection with MCMV. (**A** and **E**) Heatmaps showing the Simpson diversity index for each M38-specific (A) or IE3-specific CD8^+^ T cell repertoire (E) obtained from the peripheral blood of each mouse on days 195, 230, 265, and 302 postinfection with MCMV. (**B** and **F**) Heatmaps showing the Morisita–Horn similarity index for each pair of M38-specific (B) or IE3-specific CD8^+^ T cell repertoires (F) obtained from the peripheral blood of each mouse on days 195, 230, 265, and 302 postinfection with MCMV. (**C** and **G**) Heatbars showing the Morisita–Horn similarity index for the M38-specific (C) or IE3-specific CD8^+^ T cell repertoires (G) obtained from the peripheral blood of each mouse on days 300 and 302 postinfection with MCMV. (**D** and **H**) Heatbars showing the Morisita–Horn similarity index for the M38-specific (D) or IE3-specific CD8^+^ T cell repertoires (H) obtained from the peripheral blood and the spleen of each mouse on day 302 postinfection with MCMV.

To examine repertoire stability over time, we assigned clonotype identity at the nucleotide level, setting an exact sequence match threshold across samples. Persistent clonotypes were defined as those observed at more than one timepoint in a given mouse, and nonpersistent clonotypes were defined as those observed at only one timepoint in a given mouse. In each mouse, persistent clonotypes constituted the bulk of each inflationary CD8^+^ T cell population, irrespective of specificity (Fig. 4B, 4C). These persistent clonotypes were often numerically dominant at one or more timepoints and occurred at higher frequencies averaged per timepoint per mouse than the corresponding nonpersistent clonotypes (M38: *p* < 0.01 for mouse 2 and *p* < 0.0001 for mouse 4; IE3: *p* < 0.05 for mouse 2 and *p* < 0.01 for mouse 1, mouse 3, and mouse 4; Mann–Whitney *U* test; Fig. 4D, 4E). However, frequency variations were common over time, such that dominance patterns often shifted throughout the period of observation. It is also notable that no consistent increases or decreases in repertoire diversity or similarity were observed across timepoints (Fig. 5A, 5B, 5E, 5F). Further analyses revealed variable degrees of clonotype overlap in each mouse between specificity-matched repertoires obtained from the peripheral blood on days 300 and 302 (Fig. 5C, 5G) and between specificity-matched repertoires obtained from the peripheral blood and the spleen on day 302 (Fig. 5D, 5H). Accordingly, some of the observed fluctuations in clonotype representation across timepoints were likely due to sampling effects, and the corresponding inflationary repertoires were likely more stable than our “lower-limit” estimates (Fig. 4B, 4C).

Adoptive transfer experiments have demonstrated that a single clone primed early postinfection can be sustained during memory inflation (11, 13, 14, 31). Our data align with these observations (Fig. 1). However, it does not necessarily follow that recurrent sequences indicate clonotype survival, because nucleotide-identical precursors with the same specificity might feasibly be recruited de novo into the Ag-experienced population of inflationary CD8^+^ T cells. The frequency of any given clonotype in the naive pool can be predicted from the minimum number of nucleotide additions required to generate the corresponding TCR, which in turn correlates inversely with the likelihood of production via the V(D)J recombination machinery (38–40). On this basis, we assessed the extent to which persistent and nonpersistent clonotypes were encoded by germline genes, using the minimum number of nucleotide additions required to produce each unique sequence. In line with the possibility of ongoing recruitment from a munificent reservoir of naive precursors during the process of memory inflation, the median number of nucleotide additions required to generate persistent clonotypes was generally lower than the median number of nucleotide additions required to generate nonpersistent clonotypes (M38: *p* < 0.001 for mouse 1, *p* < 0.01 for mouse 2, *p* < 0.05 for mouse 3, and *p* < 0.0001 for mouse 4; IE3: *p* < 0.05 for mouse 1; Mann–Whitney *U* test; Fig. 4F, 4G).

### Public clonotypes are often persistent during memory inflation

**FIGURE 6.**
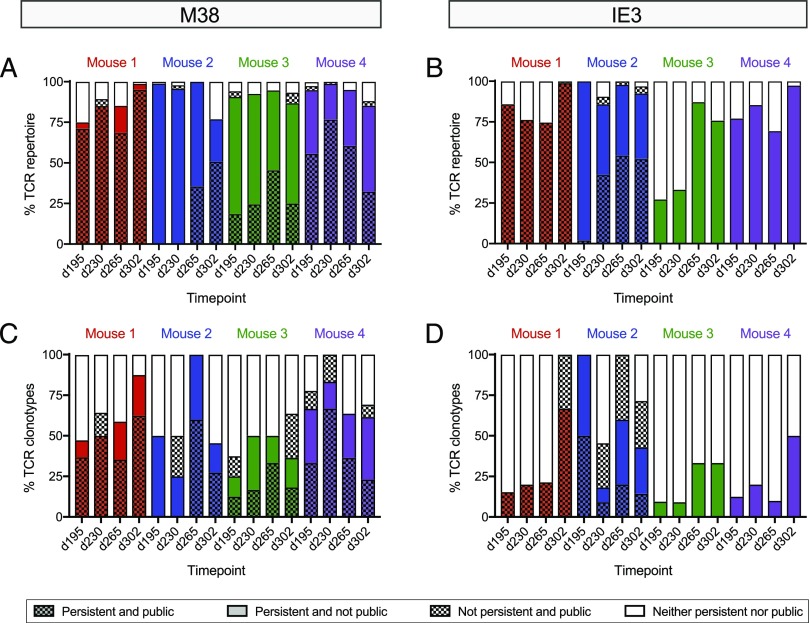
Inflationary CD8^+^ T cell repertoires often incorporate public clonotypes that persist during infection with MCMV. (**A** and **B**) Percentage of each M38-specific (A) or IE3-specific CD8^+^ T cell repertoire (B) constituted by persistent (colors) and/or public clonotypes (patterns). Data account for the frequency of each clonotype. (**C** and **D**) Percentage of unique M38-specific (C) or IE3-specific CD8^+^ T cell clonotypes (D) in each repertoire that were persistent (colors) and/or public (patterns).

Clonotypes that require no or few nucleotide additions are more likely to be present in multiple individuals than clonotypes that require multiple nucleotide additions (25, 39–43). We therefore investigated the relationship between clonotype persistence and clonotype publicity, defined at the amino acid level as an exact sequence match in more than one mouse. Large variations in the extent of clonotype sharing as a proportion of each repertoire were observed among mice and timepoints (Fig. 6A–D). Irrespective of the overall contribution of public clonotypes to each repertoire, however, persistent clonotypes were shared more commonly among mice than nonpersistent clonotypes (Fig. 6A, 6B). Moreover, clonotypes that were persistent and public constituted a greater proportion of each repertoire than clonotypes that were nonpersistent and public (Fig. 6A, 6B), and the relative predominance of clonotypes that were persistent and public (Fig. 6C, 6D) was largely attributable to higher copy numbers per sequence rather than higher numbers of unique sequences (Fig. 6A, 6B).

## Discussion

The maintenance of lifelong protection and surveillance against persistent pathogens constitutes a major challenge for the immune system. In this study, we addressed two outstanding issues pertaining to the remarkable phenomenon of memory inflation, namely the role of Ag-driven proliferation as a determinant of clonal turnover and the extent to which this process affects the overall stability of inflationary CD8^+^ T cell populations. Our data suggest that clonal expansions occur in a sporadic manner, reflecting chance encounters with cognate Ag, which in turn occur sufficiently often to sustain dominant and subdominant clonotypes within largely stable repertoires specific for inciting epitopes derived from MCMV.

A paradoxical feature of memory inflation is the apparent discrepancy between the relative paucity of viral transcripts and the disproportionately high frequencies of Ag-specific CD8^+^ T cells. It has also been proposed that effective immune surveillance further limits access to the relevant cognate epitopes, because rare viral reactivation events are detected and extinguished very rapidly by CMV-specific CD8^+^ T cells (5, 44). Our findings are consistent with this “silencing/desilencing and immune sensing” hypothesis and suggest that early “sensing” of such reactivation events occurs in a stochastic manner. However, the net effect of multiple encounters with cognate Ag ultimately seems to outweigh any constraints on epitope display at individual sites of viral reactivation, thereby providing adequate stimulation across the body as a whole to drive the process of memory inflation.

If memory inflation is driven by clonal expansions, which in turn depend on recurrent interactions with cognate Ag, then it follows that viral load must dictate the magnitude of inflationary CD8^+^ T cell populations. In line with this prediction, recent studies found that memory inflation was impaired after low-dose infection (45, 46) and enhanced by reinfection with MCMV (47). However, further work is required to assess the dynamic range of such associations, which are inextricably bidirectional and therefore most likely nonlinear over time.

In the context of memory inflation, clonal expansions are thought to originate from a precursor-like pool of memory CD8^+^ T cells, which acquire a highly differentiated phenotype as a consequence of extensive proliferation (31). The precise identity of these precursor-like memory cells nonetheless remains obscure. Potential sources of clonal replenishment include the classically defined central-memory (CD62L^+^) and effector-memory subsets (CD62L^−^), both of which are represented among inflationary CD8^+^ T cell populations (31). Each of these subsets could feasibly contribute to the process of memory inflation, because viral Ags may be encountered not only in the circulation but also in lymphoid organs (11, 13). In parenchymal tissues, however, access to viral Ags is almost certainly restricted to specialized subsets that exist in disequilibrium with the circulation and lymphoid organs, such as resident memory CD8^+^ T cells (13). This anatomical compartmentalization of the immune system likely explains an intriguing aspect of our data, namely that the ratios of congenically marked OT-I cells were distributed more unevenly in the lymph nodes and salivary glands compared with the peripheral blood and heavily vascularized organs. Inflationary CD8^+^ T cell populations may therefore be maintained primarily via homeostatic mechanisms rather than stochastic expansions in parenchymal tissues during the latent phase of infection with MCMV. In line with this notion, the long-term survival of inflationary CD8^+^ T cells in the lungs of latently infected mice was recently found to be critically dependent on IL-15 (48).

There are notable parallels between our findings and those reported from studies of HCMV. For example, the half-lives of HCMV-specific CD8^+^ T cells and MCMV-specific CD8^+^ T cells are relatively short and remarkably similar, indicating a requirement for continual exposure to Ag (29, 30). Moreover, a commonly targeted HLA-A*0201–restricted pp65 epitope derived from HCMV elicits oligoclonal CD8^+^ T cell repertoires dominated by one or two clonotypes expressing high-affinity TCRs, consistent with the notion of interclonal competition for Ag (16, 17, 21–23). Ag-specific clonotypes mobilized during early infection also persist within inflationary repertoires during chronic infection with HCMV (21, 49). However, these repertoires are not entirely static, as confirmed by our analyses in the context of MCMV (50). In addition, HCMV-specific CD8^+^ T cell populations frequently incorporate public clonotypes, some of which dominate the overall repertoire in certain individuals (16–20, 23–25).

Public clonotypes generally require fewer nucleotide additions than private clonotypes (25, 39, 40, 42, 43). In line with this essential tenet of convergent recombination, we found that persistent clonotypes were more commonly germline-encoded and more frequently public than nonpersistent clonotypes, often with a substantial presence in the corresponding inflationary CD8^+^ T cell repertoires. These observations suggest that de novo recruitment of Ag-specific precursors from the naive pool may contribute to the process of memory inflation. However, some persistent clonotypes required large numbers of nucleotide additions (*n* ≤ 9), albeit fewer than the corresponding nonpersistent clonotypes, and different near-germline-encoded clonotypes tended to persist in different mice, which argues against a probabilistic mode of replenishment. It therefore seems likely that clonotype survival is the primary determinant of repertoire stability within inflationary CD8^+^ T cell populations.

Precursor frequencies in the naive pool are known to influence the abundance and differentiation of individual clonotypes during Ag-driven immune responses (51), and competition for Ag can skew the process of memory inflation in favor of dominant CD8^+^ T cell populations (14, 15, 52). In the case of identical clonotypes, however, numerical dominance does not confer a selection advantage, as modeled in our adoptive transfer experiments with OT-I cells. A clonotype that is rare in the naive pool is therefore not automatically precluded from undergoing memory inflation. Nonetheless, the relative frequencies of congenically marked OT-I cells fluctuated substantially during the early stages of infection (up to day 35), a period characterized by rapid proliferation (11) and avidity-based competition among inflationary CD8^+^ T cell populations (14). Such dynamic interactions may therefore determine the subsequent composition of inflationary populations at equilibrium after primary infection with MCMV. The relative stability of each congenically marked population of OT-I cells during chronic infection also suggests that the latent viral reservoir is largely constant, at least in terms of Ag display. Accordingly, different viral strains with different replication and virulence profiles may elicit inflationary populations with different characteristics and greater variability over time, potentially affecting overall immune function and disease outcome.

Collectively, our data indicate that Ag encounter is infrequent at any given moment but substantial enough in aggregate to maintain an oligoclonal pool of inflationary CD8^+^ T cells during chronic infection with MCMV. These findings have potential implications for the design of next-generation vaccines based on attenuated derivatives of HCMV.

## Supplementary Material

Data Supplement
